# Supplementary figures and images for: NOTCH1 promotes the elevation of GM-CSF and IL-6 through the EZH2/STAT3 pathway to facilitate the fibrotic state of the myocardium in DLBCL
Source: PLoS One. 2025 Feb 14;20(2):e0316923. doi: 10.1371/journal.pone.0316923 (PMC11828423; doi:10.1371/journal.pone.0316923)

fig4A

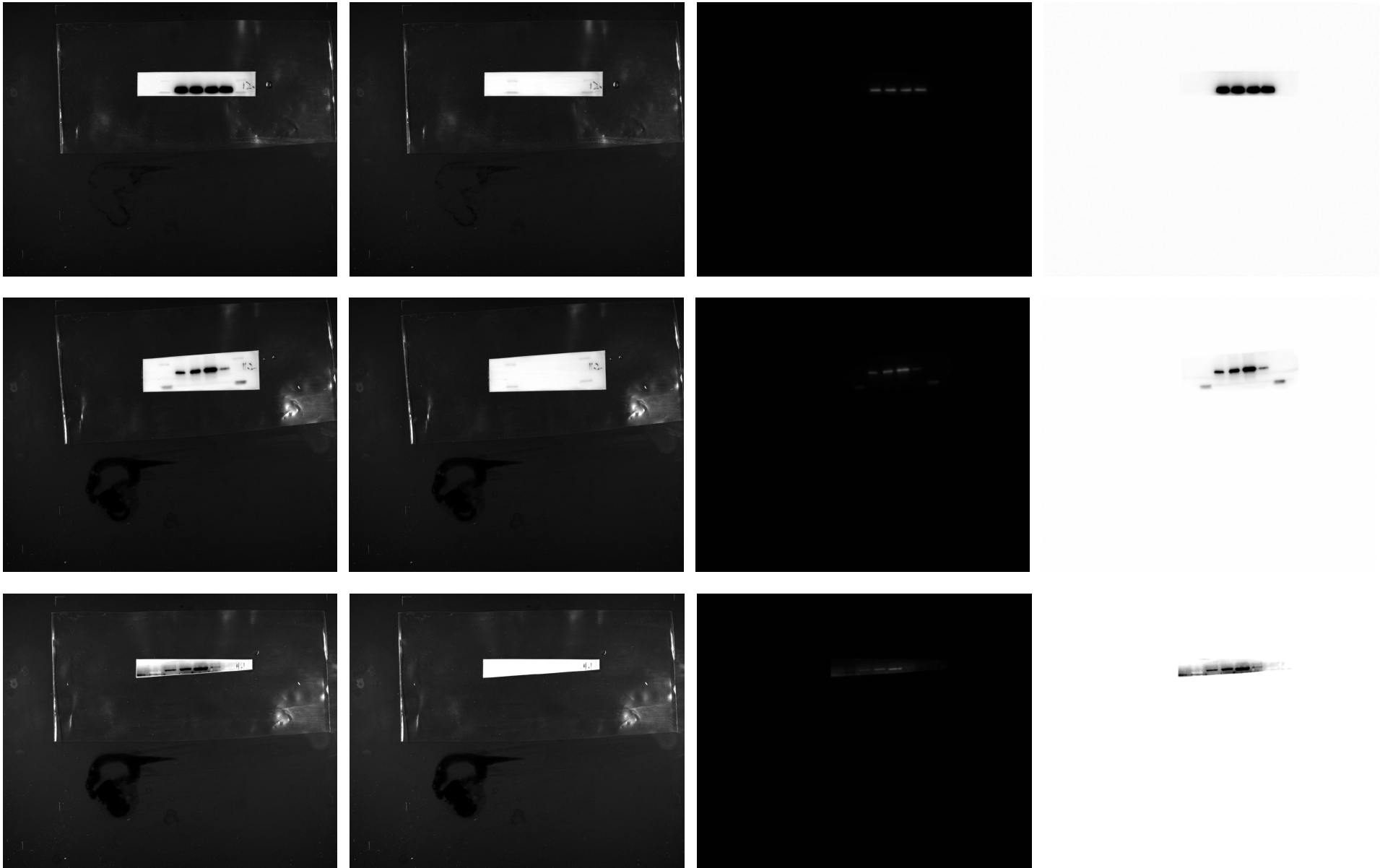

GAPDH(12)

H3K27me3

EZH2

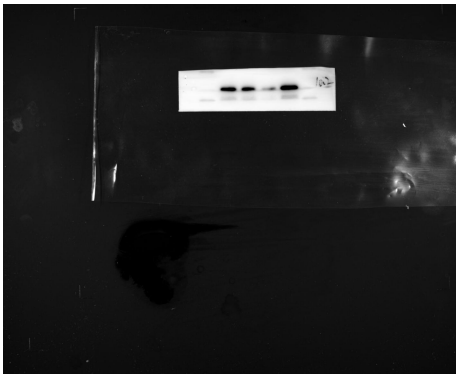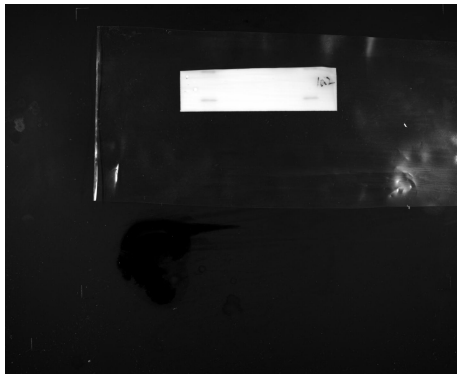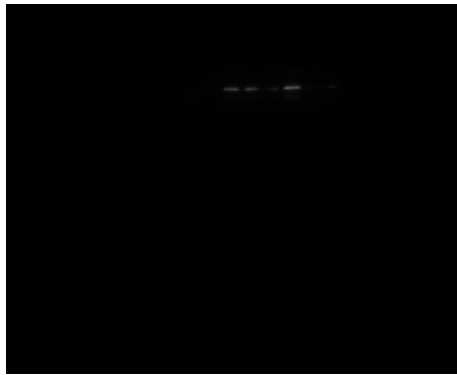

GM-CSF

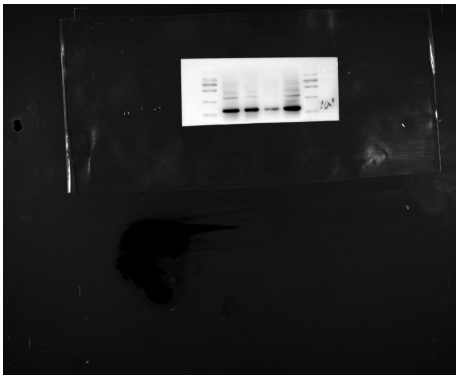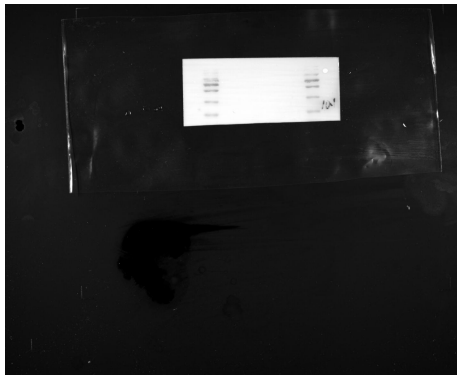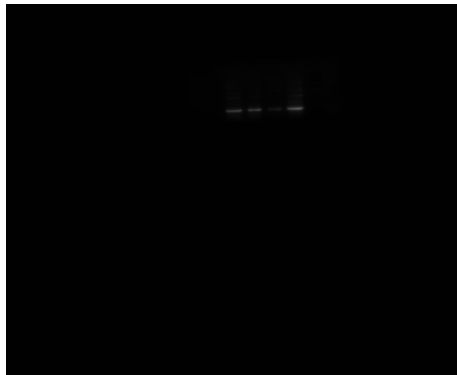

IL-6

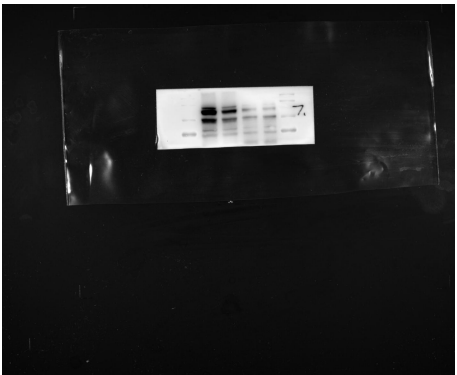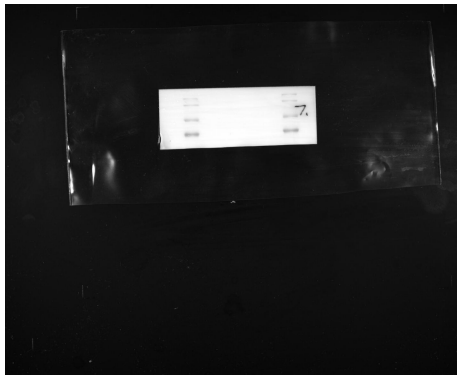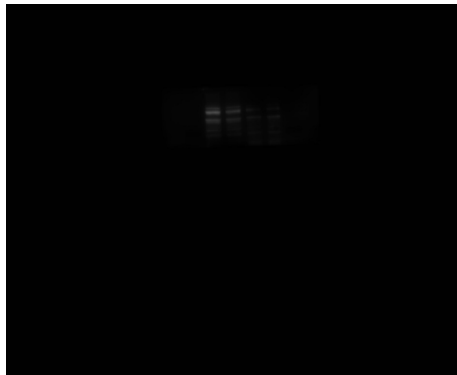

p-STAT3

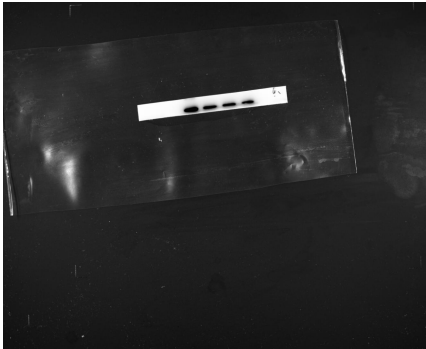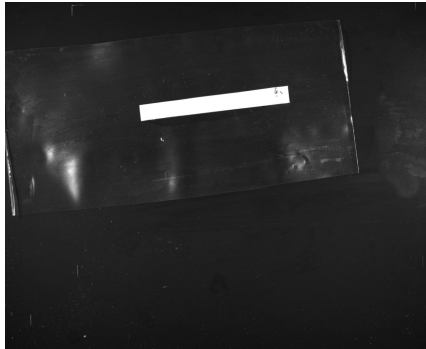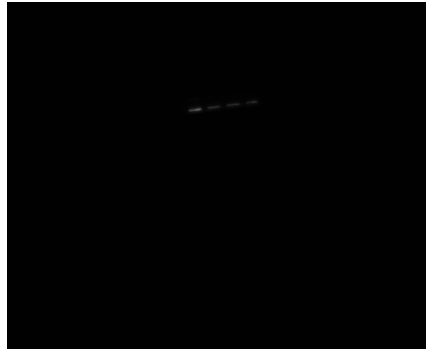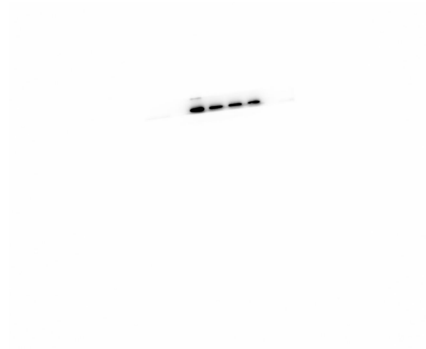

Hes1

fig5C

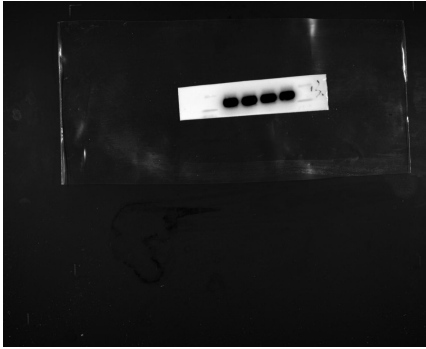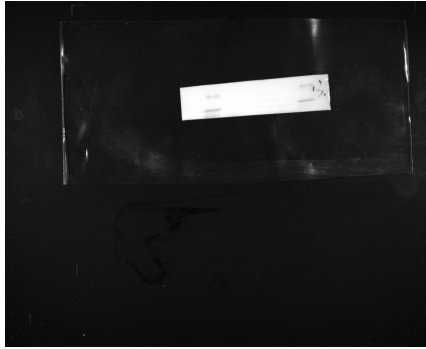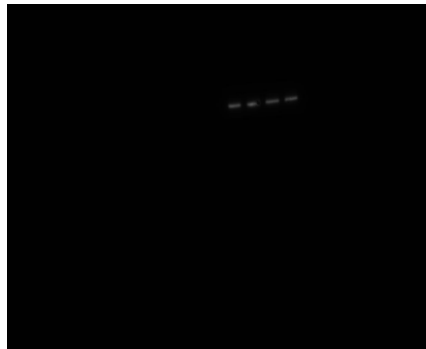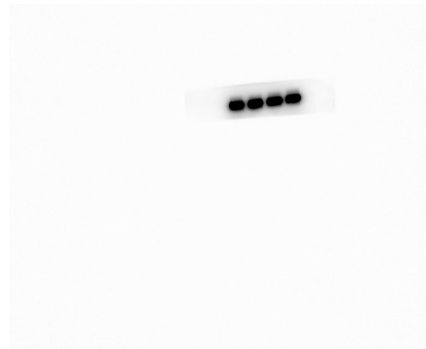

GAPDH(13

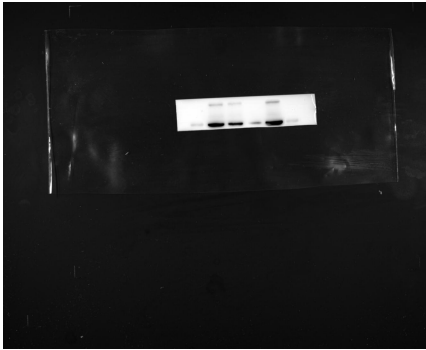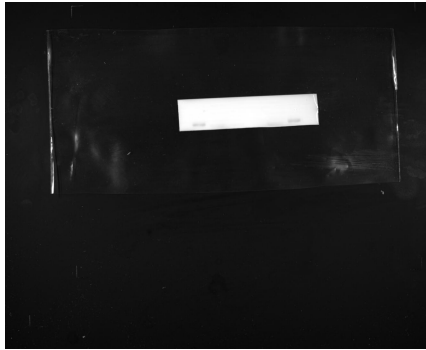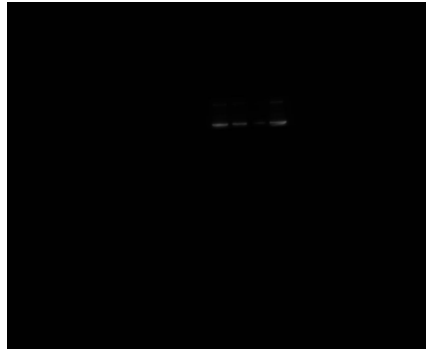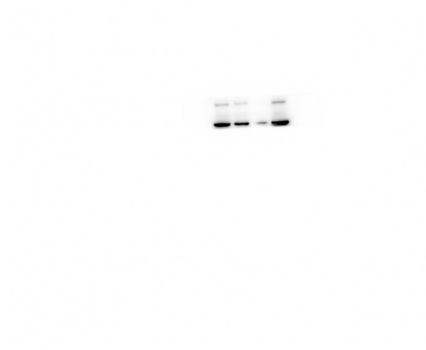

Fibronectin

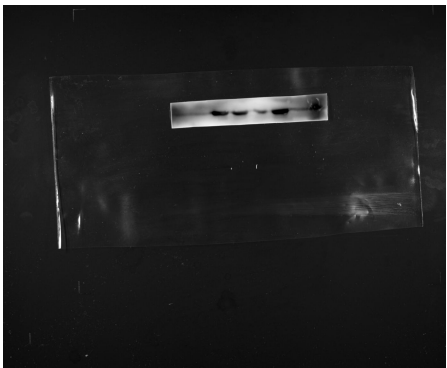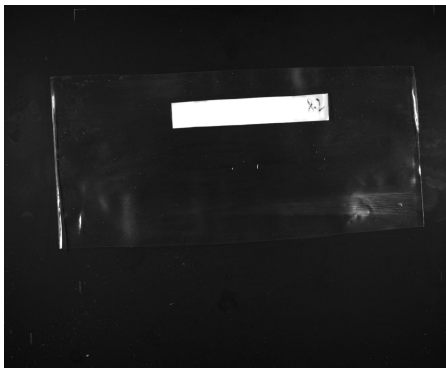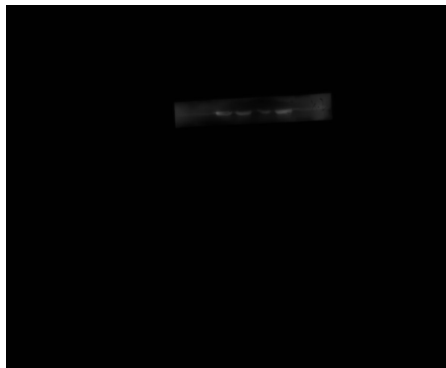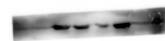

a-SMA

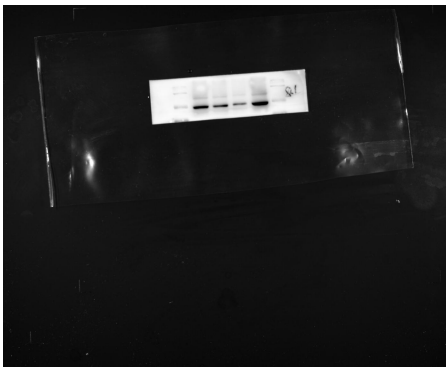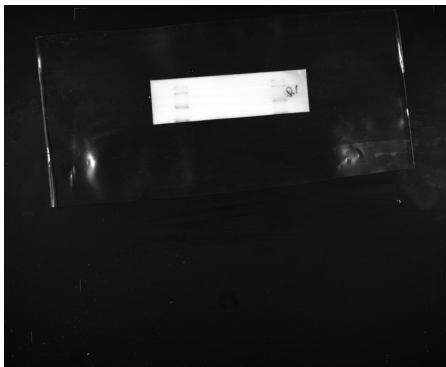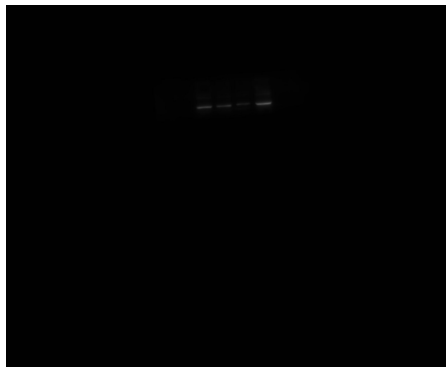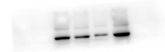

collagen-1

Supplement: S2 Table — Note: This file contains the original bands obtained in the western blot experiments generated by the study. (PDF) [file pone.0316923.s002.pdf]
