# Supplementary material for: NOTCH1 promotes the elevation of GM-CSF and IL-6 through the EZH2/STAT3 pathway to facilitate the fibrotic state of the myocardium in DLBCL
Source: PLoS One. 2025 Feb 14;20(2):e0316923. doi: 10.1371/journal.pone.0316923 (PMC11828423; doi:10.1371/journal.pone.0316923)
Supplement: Attachment — Submitted filename: Response to Reviewers.docx [file pone.0316923.s004.docx]

PONE-D-24-21738

NOTCH1 promotes the elevation of GM-CSF and IL-6 through the EZH2/STAT3 pathway to facilitate the fibrotic state of the myocardium in DLBCL

PLOS ONE

Dear Dr. Zhang,

Thank you for submitting your manuscript to PLOS ONE. After careful consideration, we feel that it has merit but does not fully meet PLOS ONE’s publication criteria as it currently stands. Therefore, we invite you to submit a revised version of the manuscript that addresses the points raised during the review process.

- Please provide necessary information in Methods and Figure Legend.
- Please have careful proofreading to reduce grammatical errors.
- Please adequately address the reviewers's concerns.

Please submit your revised manuscript by Aug 08 2024 11:59PM. If you will need more time than this to complete your revisions, please reply to this message or contact the journal office at [plosone@plos.org](mailto:plosone@plos.org). When you're ready to submit your revision, log on to https://www.editorialmanager.com/pone/ and select the 'Submissions Needing Revision' folder to locate your manuscript file.

Please include the following items when submitting your revised manuscript:

- A rebuttal letter that responds to each point raised by the academic editor and reviewer(s). You should upload this letter as a separate file labeled 'Response to Reviewers'.
- A marked-up copy of your manuscript that highlights changes made to the original version. You should upload this as a separate file labeled 'Revised Manuscript with Track Changes'.
- An unmarked version of your revised paper without tracked changes. You should upload this as a separate file labeled 'Manuscript'.

If you would like to make changes to your financial disclosure, please include your updated statement in your cover letter. Guidelines for resubmitting your figure files are available below the reviewer comments at the end of this letter.

If applicable, we recommend that you deposit your laboratory protocols in protocols.io to enhance the reproducibility of your results. Protocols.io assigns your protocol its own identifier (DOI) so that it can be cited independently in the future. For instructions see: [https://journals.plos.org/plosone/s/submission-guidelines#loc-laboratory-protocols](https://journals.plos.org/plosone/s/submission-guidelines#loc-laboratory-protocols). Additionally, PLOS ONE offers an option for publishing peer-reviewed Lab Protocol articles, which describe protocols hosted on protocols.io. Read more information on sharing protocols at <https://plos.org/protocols?utm_medium=editorial-email&utm_source=authorletters&utm_campaign=protocols>.

We look forward to receiving your revised manuscript.

Kind regards,

Meijing Wang, MD
Academic Editor
PLOS ONE

Journal Requirements:

When submitting your revision, we need you to address these additional requirements.

1. Please ensure that your manuscript meets PLOS ONE's style requirements, including those for file naming. The PLOS ONE style templates can be found at 
https://journals.plos.org/plosone/s/file?id=wjVg/PLOSOne_formatting_sample_main_body.pdf and 
https://journals.plos.org/plosone/s/file?id=ba62/PLOSOne_formatting_sample_title_authors_affiliations.pdf

Thank you for your professional advice, we have made changes to the format of the article.

2. To comply with PLOS ONE submissions requirements, in your Methods section, please provide additional information regarding the experiments involving animals and ensure you have included details on (1) methods of sacrifice, (2) methods of anesthesia and/or analgesia, and (3) efforts to alleviate suffering.

Thanks to your professional opinion, we have added Anaesthesia and Euthanasia in Mice.

3. Please note that funding information should not appear in any section or other areas of your manuscript. We will only publish funding information present in the Funding Statement section of the online submission form. Please remove any funding-related text from the manuscript.

Thanks to your professional input, we have removed the funds from the manuscript and submitted the online funds as required.

4. Thank you for stating the following financial disclosure: 
   "Funding Title: Key Technology Research Program (Hebei Provincial Health Committee)
Research Topic: Application Study of Flow Cytometry Fluorescent Immunodetection of Cytokines in Patients with Malignant Hematologic Diseases
Funding Number: 20221410"

Please state what role the funders took in the study.  If the funders had no role, please state: "The funders had no role in study design, data collection and analysis, decision to publish, or preparation of the manuscript." 
If this statement is not correct you must amend it as needed. 
Please include this amended Role of Funder statement in your cover letter; we will change the online submission form on your behalf.

Thank you for your professional opinion, we have filed that statement.

5. In the online submission form, you indicated that "All data generated or analyzed during this study are included in this published article. Additional datasets analyzed during the current study are available from the corresponding author upon reasonable request."

All PLOS journals now require all data underlying the findings described in their manuscript to be freely available to other researchers, either 1. In a public repository, 2. Within the manuscript itself, or 3. Uploaded as supplementary information.
This policy applies to all data except where public deposition would breach compliance with the protocol approved by your research ethics board. If your data cannot be made publicly available for ethical or legal reasons (e.g., public availability would compromise patient privacy), please explain your reasons on resubmission and your exemption request will be escalated for approval.

Thank you for your professional opinion, all data generated or analysed in this study are provided as Supplementary Documents and we have provided the data as Table S1.

6. PLOS requires an ORCID iD for the corresponding author in Editorial Manager on papers submitted after December 6th, 2016. Please ensure that you have an ORCID iD and that it is validated in Editorial Manager. To do this, go to ‘Update my Information’ (in the upper left-hand corner of the main menu), and click on the Fetch/Validate link next to the ORCID field. This will take you to the ORCID site and allow you to create a new iD or authenticate a pre-existing iD in Editorial Manager. Please see the following video for instructions on linking an ORCID iD to your Editorial Manager account: <https://www.youtube.com/watch?v=_xcclfuvtxQ>

Thank you for your professional input, we have provided ORCID as requested.

7. Please include your full ethics statement in the ‘Methods’ section of your manuscript file. In your statement, please include the full name of the IRB or ethics committee who approved or waived your study, as well as whether or not you obtained informed written or verbal consent. If consent was waived for your study, please include this information in your statement as well.

Thank you for your professional opinion, we have provided the ethical documents as well as the ethical statement as required.

8. Please include captions for your Supporting Information files at the end of your manuscript, and update any in-text citations to match accordingly. Please see our Supporting Information guidelines for more information: http://journals.plos.org/plosone/s/supporting-information.

Thank you for your professional opinion, we have made additional notes.

9. PLOS ONE now requires that authors provide the original uncropped and unadjusted images underlying all blot or gel results reported in a submission’s figures or Supporting Information files. This policy and the journal’s other requirements for blot/gel reporting and figure preparation are described in detail at https://journals.plos.org/plosone/s/figures#loc-blot-and-gel-reporting-requirements and https://journals.plos.org/plosone/s/figures#loc-preparing-figures-from-image-files. When you submit your revised manuscript, please ensure that your figures adhere fully to these guidelines and provide the original underlying images for all blot or gel data reported in your submission. See the following link for instructions on providing the original image data: https://journals.plos.org/plosone/s/figures#loc-original-images-for-blots-and-gels.   
In your cover letter, please note whether your blot/gel image data are in Supporting Information or posted at a public data repository, provide the repository URL if relevant, and provide specific details as to which raw blot/gel images, if any, are not available. Email us at plosone@plos.org if you have any questions.

Thank you for your professional opinion, we have submitted the original strip images for the study as well as the raw data.

[Note: HTML markup is below. Please do not edit.]

Reviewers' comments:

Reviewer's Responses to Questions

**Comments to the Author**

1. Is the manuscript technically sound, and do the data support the conclusions?

The manuscript must describe a technically sound piece of scientific research with data that supports the conclusions. Experiments must have been conducted rigorously, with appropriate controls, replication, and sample sizes. The conclusions must be drawn appropriately based on the data presented.

Reviewer #1: Partly

Reviewer #2: Partly

2. Has the statistical analysis been performed appropriately and rigorously?

Reviewer #1: No

Reviewer #2: Yes

3. Have the authors made all data underlying the findings in their manuscript fully available?

The [PLOS Data policy](http://www.plosone.org/static/policies.action#sharing) requires authors to make all data underlying the findings described in their manuscript fully available without restriction, with rare exception (please refer to the Data Availability Statement in the manuscript PDF file). The data should be provided as part of the manuscript or its supporting information, or deposited to a public repository. For example, in addition to summary statistics, the data points behind means, medians and variance measures should be available. If there are restrictions on publicly sharing data—e.g. participant privacy or use of data from a third party—those must be specified.

Reviewer #1: Yes

Reviewer #2: Yes

4. Is the manuscript presented in an intelligible fashion and written in standard English?

PLOS ONE does not copyedit accepted manuscripts, so the language in submitted articles must be clear, correct, and unambiguous. Any typographical or grammatical errors should be corrected at revision, so please note any specific errors here.

Reviewer #1: No

Reviewer #2: Yes

5. Review Comments to the Author

Please use the space provided to explain your answers to the questions above. You may also include additional comments for the author, including concerns about dual publication, research ethics, or publication ethics. (Please upload your review as an attachment if it exceeds 20,000 characters)

Reviewer #1: In this manuscript, the authors investigated the role of the NOTCH1 signaling pathway in DLBCL-related heart dysfunction. They found that NOTCH1 inhibition decreased DLBCL cell proliferation, reduced the inflammatory response, and attenuated myocardial fibrotic remodeling and infarction severity following ischemic injury. However, there are several issues that need to be addressed:

1. The manuscript contains numerous writing errors and requires significant improvement in scientific writing.
Thank you for your professional input, we have asked an English professional to touch up and revise the article.

2. Detailed methods regarding the heart ischemic injury model need to be included.
Thanks to your professional opinion, we have described in detail the detailed methodology of the cardiac ischaemic injury model.

3. The immunofluorescence staining of tissue does not appear to be of heart tissue in Figure 3c.
Thank you for your professional input, we have revised and added to this section.

4. Several important details are missing, such as the dose of the STAT3 inhibitor and the sample number. Additionally, the efficiency of shRNA is not shown.
Overall, this manuscript is not well written and lacks necessary information. The conclusions are not well supported by the provided data. Therefore, I recommend a major revision to address these issues.

Thank you for your professional opinion, we have added RT-qPCR experiments for validation and added other experiments to validate the conclusions of the article.

Reviewer #2: In this study, Zhang et al. investigated the role of the NOTCH1 signaling pathway in the pathogenesis of DLBCL-related heart disease. Through data mining and bioinformatic analysis of DLBCL and myocardial infarction GEO databases, they identified the activation of signaling pathways including NOTCH, STAT3, and EZH2, underscoring their pivotal roles in the pathogenesis of DLBCL-related heart disease. However, several issues and concerns need to be addressed:
1. The authors should justify the rationale for comparing the DLBCL and myocardial infarction GEO databases to identify signaling pathways contributing to the pathogenesis of DLBCL-related heart disease.
Thank you for your professional opinion, we investigated the signalling pathways of pathogenesis by taking the intersection of the differential genes of the DLBCL dataset GSE23501 and the myocardial infarction dataset GSE48060, and enriching the intersecting genes for pathway analysis.

2. For the bioinformatic analysis, the authors need to describe the criteria for selecting the database and how the samples were categorized into reference and observation groups.
Thank you for your professional opinion, as GEO database is chosen because it covers data from many fields such as oncology and non-oncology and has high data quality and rich data types. The datasets were grouped according to the STATUS in the dataset. The myocardial infarction dataset GSE48060 includes 31 myocardial infarction patient samples and 21 control group samples; the DLBCL dataset GSE23501 includes 69 DLBCL-related samples, which are grouped according to the STATUS in the dataset, and the samples are classified into 7 reference groups and 62 observation groups.

3. The co-expression scatter plots in Figure 1 G-J do not support the claimed correlations between RBPJ and EZH2, EZH2 and H3K27, and STAT3 and JMD3, except NOTCH1 and RBPJ.
Thanks to your professional opinion, we have modified the correlation analysis for NOTCH1 and STAT3, NOTCH1 and EZH2, the correlation coefficient of NOTCH1 and STAT3 is 0.646, and the correlation coefficient of NOTCH1 and EZH2 is -0.543.

4. In Figure 5 A-D, the results for Static treatment alone should be presented to conclusively determine the synergistic effects of NOTCH and STAT3 signaling inhibition on fibroblast growth.
Thanks to your professional advice, we have added experiments in this section using STAT3 agonists and inhibitors to treat the study cells individually and set up a control group to determine the synergistic effect of NOTCH and STAT3 signalling inhibition on fibroblast growth.

5. The manuscript appears to be prepared hastily, containing numerous grammatical errors. Additionally, the figure legends lack detail and do not always correspond to the figures presented. For instance, in the legend for Figure 1, there is no distinction between panels 1A-a and 1A-b; there is no description for Figures 1D and 1E. Furthermore, Figure 1C contains two panels both labeled as Figure 1C-a. Also, many abbreviations are used without being defined upon first use.

Thank you for your professional input, we have revised and detailed the pictures and legends.

6. PLOS authors have the option to publish the peer review history of their article ([what does this mean?](https://journals.plos.org/plosone/s/editorial-and-peer-review-process#loc-peer-review-history)). If published, this will include your full peer review and any attached files.
Thank you for your professional opinion, we choose no.

If you choose “no”, your identity will remain anonymous but your review may still be made public.


**Do you want your identity to be public for this peer review?** For information about this choice, including consent withdrawal, please see our [Privacy Policy](https://www.plos.org/privacy-policy).

Reviewer #1: No

Reviewer #2: No

[NOTE: If reviewer comments were submitted as an attachment file, they will be attached to this email and accessible via the submission site. Please log into your account, locate the manuscript record, and check for the action link "View Attachments". If this link does not appear, there are no attachment files.]

While revising your submission, please upload your figure files to the Preflight Analysis and Conversion Engine (PACE) digital diagnostic tool, <https://pacev2.apexcovantage.com/>. PACE helps ensure that figures meet PLOS requirements. To use PACE, you must first register as a user. Registration is free. Then, login and navigate to the UPLOAD tab, where you will find detailed instructions on how to use the tool. If you encounter any issues or have any questions when using PACE, please email PLOS at [figures@plos.org](mailto:figures@plos.org). Please note that Supporting Information files do not need this step.
